# Clinical characteristics and spectrum of *NF1* mutations in 12 unrelated Chinese families with neurofibromatosis type 1

**DOI:** 10.1186/s12881-018-0615-8

**Published:** 2018-06-18

**Authors:** Bin Mao, Siyu Chen, Xin Chen, Xiumei Yu, Xiaojia Zhai, Tao Yang, Lulu Li, Zheng Wang, Xiuli Zhao, Xue Zhang

**Affiliations:** 1 Department of Medical Genetics, Institute of Basic Medical Sciences, Chinese Academy of Medical Sciences & School of Basic Medicine, Peking Union Medical College, Beijing, 100005 China; 2 Department of Obstetrics and Gynecology, the First Affiliated Hospital of Hebei North University, Zhangjiakou, 075061 China

**Keywords:** Neurofibromatosis type 1, The *NF1* gene, Clinical manifestations, Chinese

## Abstract

**Background:**

Neurofibromatosis type 1 (NF1) is a common autosomal dominant disorder caused by a heterozygous germline mutation in the tumor suppressor gene *NF1*. Because of the existence of highly homologous pseudogenes, the large size of the gene, and the heterogeneity of mutation types and positions, the detection of variations in NF1 is more difficult than that for an ordinary gene.

**Methods:**

In this study, we collected samples from 23 patients among 46 study participants from 12 unrelated Chinese families with NF1. We used a combination of Sanger sequencing, targeted next-generation sequencing, and multiplex ligation-dependent probe amplification to identify potential mutations of different types.

**Results:**

Seven recurrent mutations and four novel mutations were identified with the aforementioned methods, which were subsequently confirmed by either restriction fragment length polymorphism analysis or Sanger sequencing. Truncating mutations accounted for 73% (8/11) of all mutations identified. We also exhaustively investigated the clinical manifestations of NF1 in patients via acquired pathography, photographs and follow-up. However, no clear genotype–phenotype correlation has been found to date.

**Conclusion:**

In conclusion, the novel mutations identified broaden the spectrum of *NF1* mutations in Chinese; however, obvious correlations between genotype and phenotype were not observed in this study.

**Electronic supplementary material:**

The online version of this article (10.1186/s12881-018-0615-8) contains supplementary material, which is available to authorized users.

## Background

Neurofibromatosis type 1 (NF1; MIM: 162200) is one of the most common autosomal dominant inherited diseases with an incidence of 1 in 2500–3000 individuals [1]. Caused by a germline heterozygous mutation in the tumor suppressor gene neurofibromin 1 (*NF1*; MIM: 613113) located on chromosome 17q11.2, NF1 is characterized by typical café-au-lait spots and cutaneous neurofibromas [2]. Individuals with NF1 are predisposed to plexiform neurofibromas, axillary and inguinal freckling, Lisch nodules of the iris, benign and malignant tumors, and renal artery stenosis, among a list of other abnormalities [3]. Although NF1 is a classical monogenic disease with complete penetrance by adulthood, clinical symptoms may vary in patients who come from the same family, or even for the same patient at different life stages. Complex though the clinical manifestations of patients may be, individuals in this study were diagnosed as NF1 only when they met two or more of the National Institutes of Health Diagnostic Criteria for NF1 [4].

*NF1* is one of the largest known genes with a genomic size of 282 kb, consisting of 57 constitutive exons and three alternatively spliced exons [5]. Owing to its extremely frequent incidence of mutation (circa 1 in 10,000 gametes per generation) without obvious mutational hot spots, over 2600 *NF1* mutations have hitherto been reported in the Human Gene Mutation Database (HGMD). Single nucleotide substitutions and small deletions (20 bp or less) account for 71% of currently known mutations. Moreover, approximately half of all NF1 cases are de novo mutations [6]. In addition, the large size of the *NF1* gene, the existence of homologous pseudogenes dispersed on other chromosomes [7], the diversity of mutation types and positions, and the great variety of lesions make traditional mutation detection in patients with NF1 a complicated, time-consuming and laborious process [8]. With the superiority of being high throughput and its rapidity, the next-generation sequencing can make up for any deficiency in the single Sanger sequencing method to some extent. In addition, multiplex ligation-dependent probe amplification (MLPA) for the detection of copy number was incorporated in our methods. Hence, we adopted various approaches such as Sanger sequencing, targeted next-generation sequencing, and MLPA so as to overcome challenges in the detection of *NF1* mutations in patients.

Despite several reports with regard to genotype–phenotype correlations in patients [9–11], the underlying causes of sophisticated clinical manifestations among patients have not yet been elucidated [12]. Nevertheless, the causes of polymorphisms in genotype–phenotype correlations may be assigned to modifier genes, gender, loss of heterozygosity (based on the two-hit hypothesis) [13], tumor microenvironment, and heterogeneity in the regulation of signaling pathways [3]. Consequently, it is of great significance to identify the causative mutation and assess the prognosis of NF1 patients, if genotype–phenotype correlations can be clarified, before the onset of symptoms.

In brief, we examined 12 non-consanguineous Chinese families from which patients were diagnosed with NF1. A molecular diagnosis and clinical characterization of NF1 patients were undertaken to identify causative mutations and evaluate any correlations between genotype and phenotype.

## Methods

### Patients

**Fig. 1:**
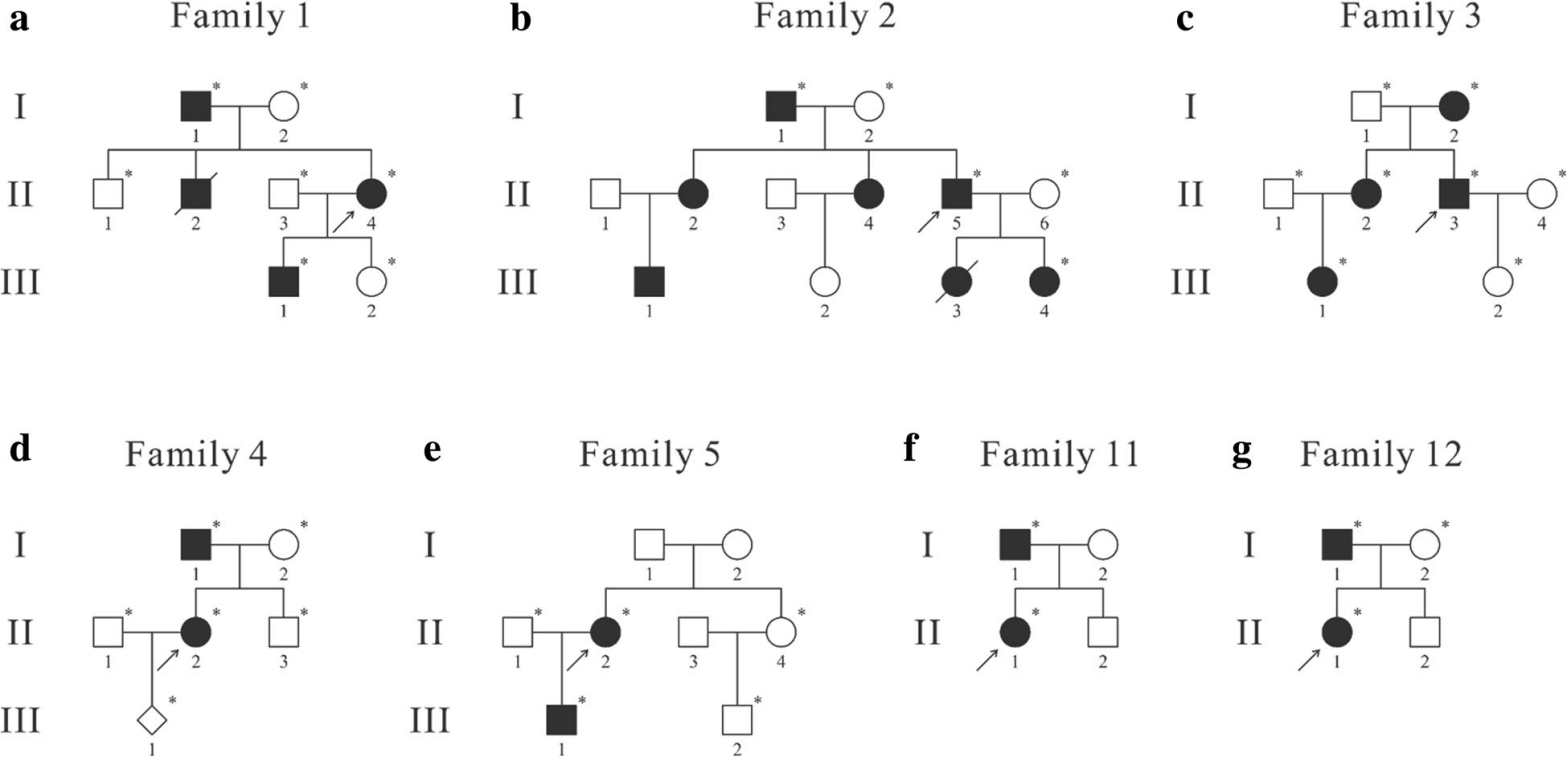
Pedigrees of families with positive family histories. The arrows indicate the probands in each family. The asterisks denote that peripheral blood samples of individuals had been acquired. **a**-**g**: Pedigrees of Families 1–5 and 11–12

We studied 12 unrelated families with NF1 from different regions in China that included seven cases with positive family histories (Families 1–5 and 11–12; Fig. 1) and five sporadic cases (Families 6–10; Additional file 1: Figure S1), including 23 patients and 23 unaffected individuals. Peripheral blood samples of all 46 participants, as well as clinical data and photographs of patients, were obtained after written informed consent from all participants and from parents or legal guardians of children under the age of 18. Long-term follow-up was also performed with several contactable patients to evaluate progression of the disease. Age of patients was recorded at their last visit in this study. This study was approved by the Institutional Review Board (IRB) of the Institute of Basic Medical Sciences, Chinese Academy of Medical Sciences, Beijing, China (015–2015).

### Sanger sequencing

For earlier probands in Families 1–8 and other study participants in Families 4–12, the identification and verification of mutations were carried out with conventional Sanger sequencing. Genomic DNA was extracted by a traditional proteinase K and phenol/chloroform method. Genomic DNA and cDNA reference sequences of *NF1* (hg19; NM_000267.3) were downloaded from the University of California, Santa Cruz (UCSC) Genome Browser. Primers were designed via Primer Premier 5 (version 5.00; PREMIER Biosoft, Palo Alto, CA, USA) to amplify and sequence exons and flanking intronic regions of *NF1* (Additional file 1: Table S1). The specificity of the primers was checked using the UCSC Genome Browser BLAT and *In-Silico* PCR online tools. Sequencing data was analyzed using CodonCode Aligner (version 6.0.2.6; CodonCode, Centerville, MA, USA).

### Targeted next-generation sequencing

The mutation identification of later probands in Families 9–12 was performed through targeted next-generation sequencing. A NimbleGen capture panel (Roche, Basel, Switzerland) was designed and assessed to detect potential variants in the probands. The capture panel comprised 10,308 bp that covered all exons together with flanking intronic regions (± 15 bp) of the *NF1* and *NF2* genes.

Genomic DNA was extracted using a QIAamp DNA Blood Midi Kit (QIAGEN, Hilden, Germany) in accordance with the manufacturer’s instructions. Genomic DNA was then fragmented for the paired-end library (200–250 bp) using an ultrasonicator LE220 (Covaris, Woburn, MA, USA). The library was enriched through array hybridization at 47 °C for 64–72 h, with elution and post-capture amplification afterwards. The library was then inspected using a 2100 Bioanalyzer (Agilent, Santa Clara, CA, USA) and ABI StepOne (Thermo Fisher Scientific, Waltham, MA, USA) to estimate the size, concentration, and magnitude of the enrichment of the reads.

After the assessment of read quality, captured library sequencing was implemented on a HiSeq2500 System high-throughput sequencing system (Illumina, San Diego, CA, USA) for 90 cycles per read following the manufacturer’s instructions. Image analysis, error estimation and base calling were performed with Pipeline software (version 1.3.4; Illumina) to generate raw data.

### MLPA

For the proband in whom a causative mutation was not identified by Sanger sequencing or targeted next-generation sequencing, P081 (version C1) and P082 (version C1) MLPA probemixes (MRC-Holland, Amsterdam, the Netherlands) were applied to detect copy number variation in conformity with the manufacturer’s instructions. Capillary electrophoresis results of MLPA samples were analyzed by Coffalyser.Net software (version 140,721.1958; MRC-Holland).

### Bioinformatics analysis

The raw data from targeted next-generation sequencing was screened by filtering criteria to remove low-quality and contaminated reads [14]. Reads were then aligned to the human genome reference (hg19) by a Burrows Wheeler Aligner–backtrack software package [15]. The sequencing coverage and depth of the target region, single nucleotide variant (SNV) and indel calling, were analyzed after alignment. Software Short Oligonucleotide Analysis Package–snp (version 1.03; Beijing Genomics Institute, Beijing, China) [16] and Sequence Alignment/Map tools (version 1.4) [17] were used to detect SNVs and indels, respectively. After acquisition of the allele frequency from the UCSC Genome and ExAC Browsers database to eliminate the possibility of single nucleotide polymorphism (SNP), we consulted the HGMD and other references to study relevant reports about screened variants in all probands.

For missense variants, the online tools Polymorphism Phenotyping v2 (PolyPhen-2) [18], Scale-Invariant Feature Transform (SIFT) [19], and Mutation Taster [20] were utilized to predict the pathogenicity of each variant. Multiple sequence alignment and conservative analysis were performed by ClustalX software (version 2.1; Conway Institute, University College Dublin, Dublin, Republic of Ireland). The amino acid sequences of human neurofibromin (NP_000258.1) and that of 11 different vertebrates were obtained from the National Center for Biotechnology Information (NCBI) protein database (FASTA format). For frame shift variants (small deletions and single nucleotide duplication), DNAMAN (version 5.2.2; Lynnon Biosoft, San Ramon, CA, USA) was used to predict how the reading frame was interrupted and to calculate the number of nucleotides before a premature stop codon.

### Restriction fragment length polymorphism

Restriction fragment length polymorphism (RFLP) was used, together with nested PCR and restriction endonuclease, to discriminate between genotypes of patients and that of unaffected individuals in Families 1–3 with larger pedigrees. In addition to the primers used for Sanger sequencing, nested PCR primers were designed to enhance the specificity of small DNA fragments or to introduce a mismatch nucleotide to create a new restriction site (Additional file 1: Table S1). Sequence differences between wild-type and mutant alleles resulted in the gain or loss of a restriction site that led to size differences between amplicons of different alleles after the restriction endonuclease reaction. The restriction endonucleases (New England Biolabs, Ipswich, MA, USA) *Taq*^α^ I (restriction site: T|CGA), *Alu* I (restriction site: AG|CT), and *Sac* II (restriction site: CCGC|GG) were applied to Families 1, 2, and 3, respectively. Polyacrylamide gel electrophoresis (PAGE) using an 8% neutral polyacrylamide gel was then performed to separate DNA fragments of different sizes. Electrophoresis conditions included 1 × TBE as electrophoresis buffer and a constant voltage of 350 V for 3–5 h. Silver staining was used for the final step of the chromogenic reaction.

## Results

### Clinical manifestations

**Fig. 2:**
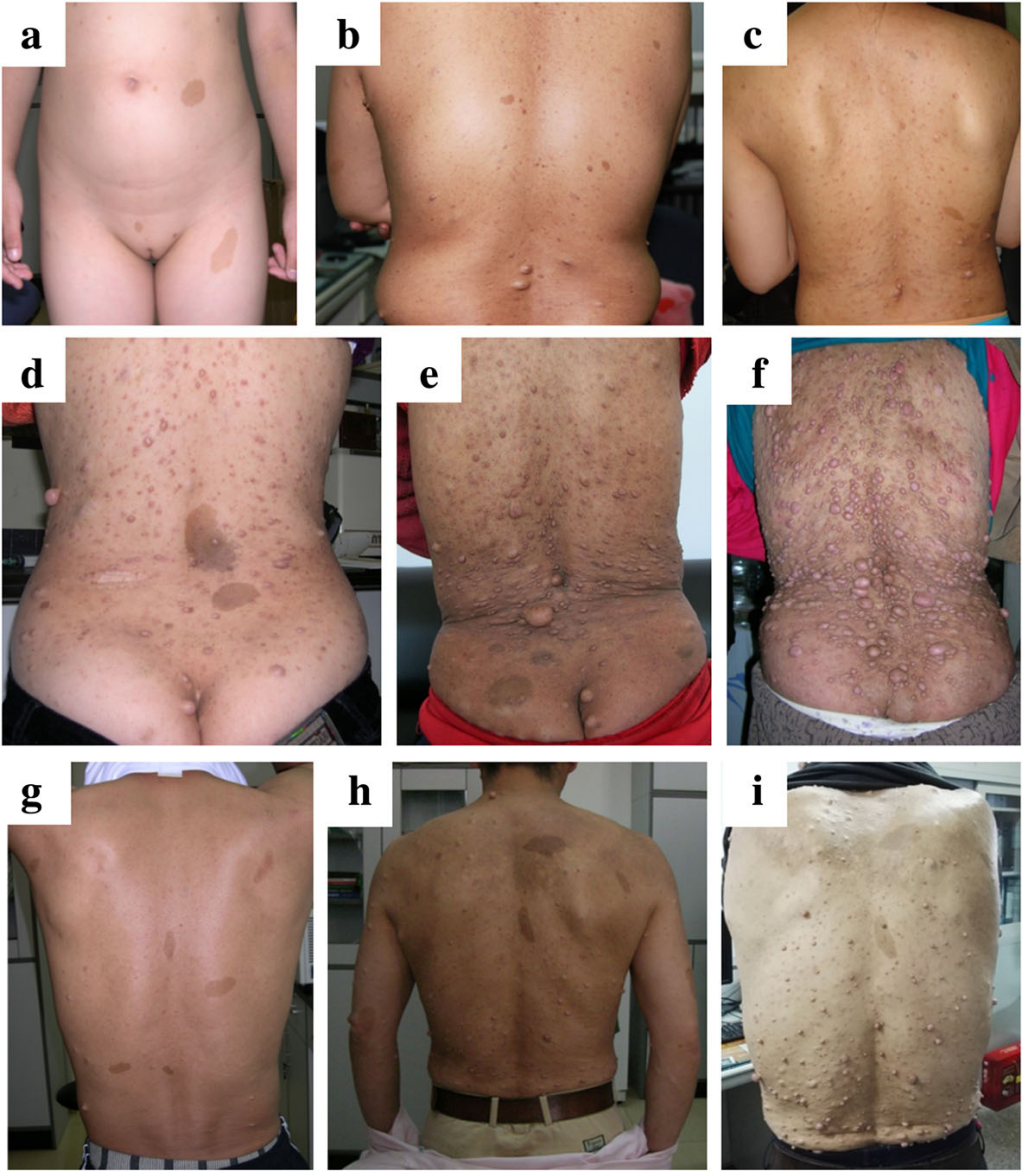
Clinical signs of NF1 in several patients. **a**: Café-au-lait spots on the abdomen and left leg of prepubertal female Patient 17; **b**–**i**: Café-au-lait spots and neurofibromas on the back of postpubertal females without (Patients 11 and 20) or with (Patients 18, 1, and 10) histories of pregnancy, and postpubertal males (Patients 4, and 7 in 2007 and 2017, respectively)

A general description of the clinical manifestations of 23 NF1 patients are listed in Table 1, with typical symptoms shown in Fig. 2. It is regrettable that on account of advanced age, geographical distances, or for personal reasons, the detailed clinical data and photographs of six patients (Patients 2, 6, 8, 9, 14 and 21) were not available except for their peripheral blood samples. Of the readily obtained clinical symptoms of the remaining 17 patients, café-au-lait spots were observed in all 17 patients and were found spotted in one or more skin regions immediately after birth in Patients 1, 5, 7, 11, 15, 16, 17, 19, 20 and 22. Axillary or inguinal freckling was the second most common phenotype that accounted for 94% (16/17) of cases. Additionally, 13 (76%) patients suffered from cutaneous neurofibromas, six (35%) of which were also found to have plexiform neurofibromas. In terms of the location of skin lesions, these were present on the trunk in all 17 (100%) patients, followed by limbs (upper and lower limbs) in 11 (65%), neck in seven (41%) and face (chin, forehead and cheek) in four (24%).

**Table 1:** Available clinical symptoms of NF1 patients

Family number	Family history	Patient number	Pedigree number	Age	Gender	Number of café-au-lait spots	Largest diameter of café-au-lait spots	Number of NFs	Largest Diameter of NFs	Plexiform NFs	Axillary/Inguinal Freckling	Location of Skin Lesions
1	Yes	1	II-4	35–39	Female	7	5.5 cm	300–400	3.5 cm	Yes	Yes	Trunk, Neck, Chin, Forehead
		2	III-1	15–19	Male	N/A						
		3	I-1	75–79	Male	5	4 cm	200–300	2 cm	Yes	Yes	Trunk, Neck, Chin, Cheek
2	Yes	4	II-5	25–29	Male	13	5.5 cm	10–20	1.5 cm	No	Yes	Trunk
		5	III-4	0–4	Female	3	2.5 cm	0	–	No	No	Trunk, Lower Limbs
		6	I-1	55–59	Male	N/A						
3	Yes	7	II-3	50–54	Male	9	6 cm	300–400	2 cm	Yes	Yes	Trunk, Limbs, Neck, Chin
		8	II-2	40–44	Female	N/A						
		9	III-1	10–14	Female	N/A						
		10	I-2	65–69	Female	3	5 cm	1000–2000	3 cm	Yes	Yes	Trunk, Limbs
4	Yes	11	II-2	25–29	Female	13	4.5 cm	10–20	2 cm	No	Yes	Trunk, Lower Limbs
		12	I-1	45–49	Male	14	5 cm	20–30	2 cm	No	Yes	Trunk, Limbs
5	Yes	13	II-2	45–49	Female	12	4 cm	50–100	1.5 cm	No	Yes	Trunk, Lower Limbs, Neck
		14	III-1	10–14	Male	N/A						
6	No	15	II-1	15–19	Female	28	7 cm	0	–	No	Yes	Trunk, Upper Limbs
7	No	16	II-1	40–44	Female	14	4.5 cm	50–100	1.5 cm	Yes	Yes	Trunk, Limbs
8	No	17	II-1	15–19	Female	34	5 cm	0	–	No	Yes	Trunk, Limbs
9	No	18	II-1	30–34	Female	9	7.5 cm	200–300	2.5 cm	Yes	Yes	Trunk
10	No	19	II-1	30–34	Female	7	3.5 cm	0	–	No	Yes	Trunk, Neck
11	Yes	20	II-1	25–29	Female	16	4 cm	100–200	1.5 cm	No	Yes	Trunk, Neck
		21	I-1	45–49	Male	N/A						
12	Yes	22	II-1	25–29	Female	8	6.5 cm	100–200	1 cm	No	Yes	Trunk, Limbs, Neck, Forehead
		23	I-1	55–59	Male	6	3 cm	200–300	1.5 cm	No	Yes	Trunk, Upper Limbs

*NFs* neurofibromas, *N/A* not available

### NF1 mutation Spectrum

**Fig. 3:**
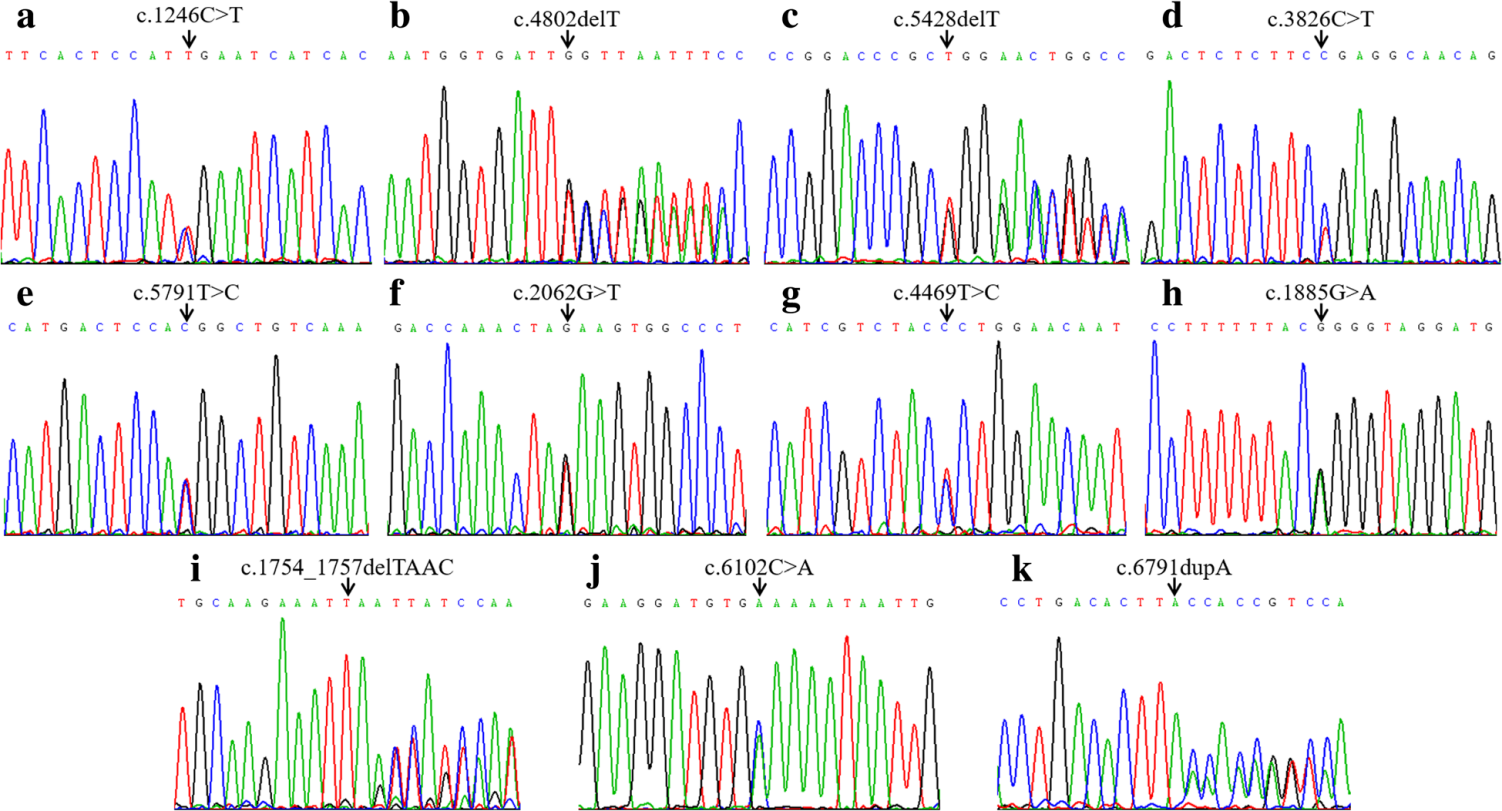
Mutations identified in the probands of Families 1–11. Mutations detected by Sanger sequencing: **a**: Patient 1, c.1246C>T; **b**: Patient 4, c.4802delT; **c**: Patient 7, c.5428delT; **d**: Patient 11, c.3826C>T; **e**: Patient 13, c.5791T>C; **f**: Patient 15, c.2062G>T; **g**: Patient 16, c.4469T>C; **h**: Patient 17 c.1885G>A; **i**: Patient 18, c.1754_1757delTAAC; **j**: Patient 19, c.6102C>A; **k**: Patient 20, c.6791dupA

We identified 11 different germline mutations in probands from the 12 families mentioned above; these were located in different exons across the gene (Fig. 3 and Table 2). In other words, a mutational hot spot was not detected according to our findings. Remarkably, the proportion of truncating mutations approached 73% (8/11), and was composed of four (36%) nonsense mutations (c.1246C>T, p.R416*; c.2062G>T, p.E688*; c.3826C>T, p.R1276*; c.6102C>A, p.C2034*), three (27%) small deletions (c.4802delT, p.L1601Cfs*2; c.5428delT, p.W1810Gfs*32; c.1754_1757delTAAC, p.T586Vfs*18), and one (9%) single nucleotide duplication (c.6791dupA, p.Y2264*). In addition, the remaining three (27%) variants were all missense mutations (c.5791T>C, p.W1931R; c.4469T>C, p.L1490P; c.1885G>A, p.G629R). For the proband in Family 12 (Patient 22) in whom a causative mutation was not identified by targeted next-generation sequencing, an abnormal copy number was not detected in either Patient 22 or her father (Patient 23) using MLPA (Additional file 1: Figure S2).

**Table 2:** NF1 mutations identified in this study

Family Number	Mutation Position	Nucleotide Change	Amino Acid Change	Mutation Type	References
1	Exon 11	c.1246C>T	p.R416*	Nonsense	Reported^a^
2	Exon 36	c.4802delT	p.L1601Cfs*2	Deletion	Novel
3	Exon 37	c.5428delT	p.W1810Gfs*32	Deletion	Novel
4	Exon 28	c.3826C>T	p.R1276*	Nonsense	Reported
5	Exon 39	c.5791T>C	p.W1931R	Missense	Reported
6	Exon 18	c.2062G>T	p.E688*	Nonsense	Novel
7	Exon 33	c.4469T>C	p.L1490P	Missense	Reported
8	Exon 17	c.1885G>A	p.G629R	Missense	Reported
9	Exon 16	c.1754_1757delTAAC	p.T586Vfs*18	Deletion	Reported
10	Exon 41	c.6102C>A	p.C2034*	Nonsense	Novel
11	Exon 45	c.6791dupA	p.Y2264*	Duplication	Reported

^a^The mutation has been reported in the Human Gene Mutation Database (HGMD; Professional 2016.4)

Furthermore, the mutations we identified in Families 2 (c.4802delT, p.L1601Cfs*2), 3 (c.5428delT, p.W1810Gfs*32), 6 (c.2062G>T, p.E688*), and 10 (c.6102C>A, p.C2034*), respectively, as far as we know, have not been reported previously. It is noteworthy that the four novel mutations are all truncating mutations that are generally considered to introduce a premature stop codon in the reading frame.

### Mutation verification in the families

**Fig. 4:**
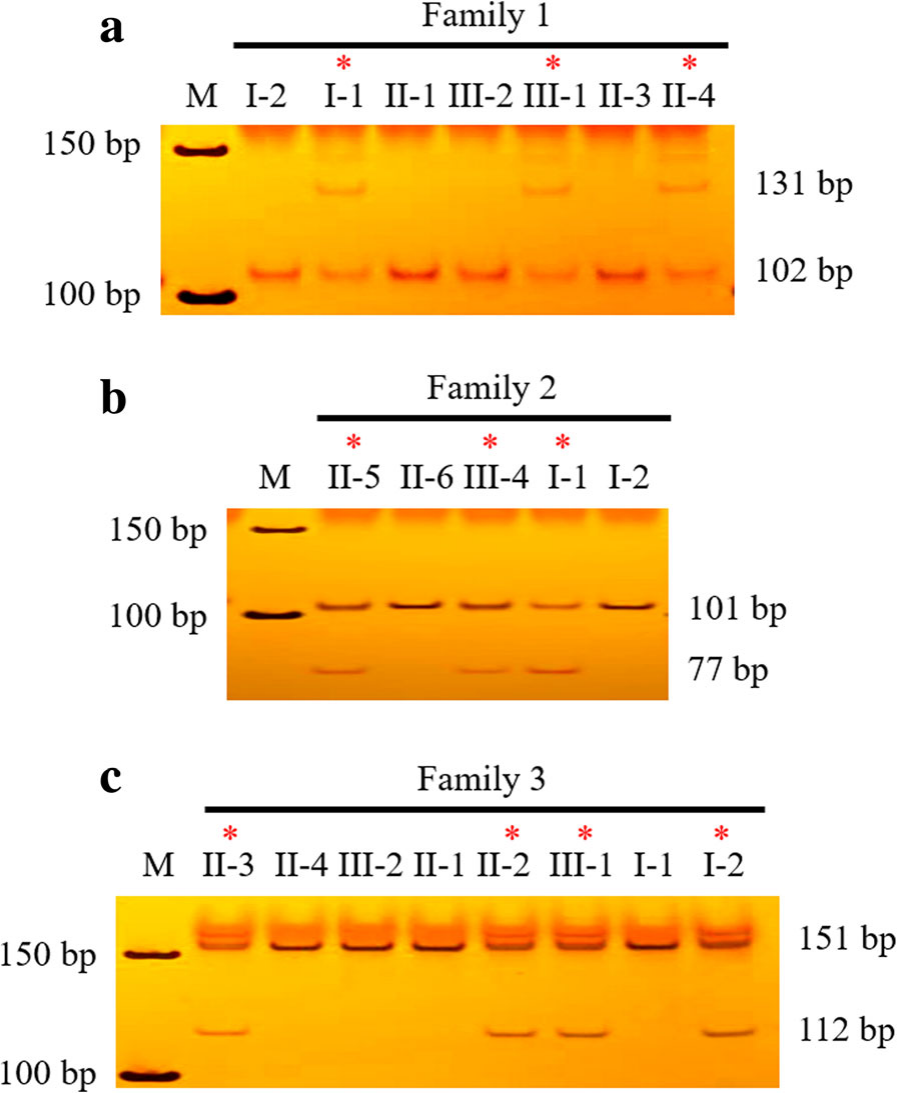
Neutral polyacrylamide gel electrophoresis *(*PAGE) of DNA samples from all study participants in Families 1–3. A 50-bp DNA Ladder was used as a marker (lane M). Red asterisks denote affected individuals. The numbers on the left and right of the figures represent the size of markers and DNA fragments, respectively. **a**: Neutral PAGE of DNA samples from participants in Family 1. The wild-type allele had a *Taq*^α^ I restriction site, and was therefore digested into 29-bp (not shown) and 102-bp fragments; **b**: Neutral PAGE of DNA samples from participants in Family 2. The mutation c.4802delT resulted in a gain of the *Alu* I restriction site after the forward primer (*NF1*-family 2F; Additional file 1: Table S1) introduced a mismatch nucleotide in its 3′ end, and the mutant allele was subsequently digested into 24-bp (not shown) and 77-bp fragments; **c**: Neutral PAGE of DNA samples from participants in Family 3. The mutant allele was digested into 39-bp (not shown) and 112-bp fragments by *Sac* II since the deletion of thymidylate produced a new restriction site

For individuals in Families 1–3, a nested PCR–restriction endonuclease reaction–neutral PAGE method was adopted. All patients in the three families were found to carry the same mutation as that of the probands, and were heterozygotes for mutant alleles, while all unaffected individuals only had wild-type alleles (Fig. 4).

Sanger sequencing was performed to verify mutations in individuals of Families 4–11. It turned out that other patients in the families all carried mutations identical to that of the probands, while unaffected individuals were all homozygotes for wild-type alleles (data available on request).

### Genotype–phenotype correlations

Patients who had rather serious clinical manifestations (from Families 1, 3 and 9) were all heterozygotes for the truncating mutation. However, other patients with milder symptoms carried similar truncating or missense mutations. Furthermore, patients who carried the same mutation within the one family exhibited diverse clinical symptoms. For example, Patient III-3 in Family 2 (samples not available), whose father (II-5; Patient 4) merely manifested moderate symptoms, died of acute infantile spasm 10.5 months after birth as a complication of NF1, while her affected sister (III-4; Patient 5) fortunately survived. Moreover, the phenotype of Patient 1 was slightly more severe than that of her father (Patient 3).

## Discussion

In this study, we utilized a synthetic method of Sanger sequencing, targeted next-generation sequencing, and MLPA to detect potential mutations in patients. We also investigated the clinical presentations of each patient with NF1 to elucidate the factors associated with the severity of the disease phenotype.

As a consequence, 11 different mutations scattered in different exons of *NF1* were identified in 12 unrelated Chinese families with NF1, suggesting a positive detection rate of 92% (11/12). Though no mutational hot spot was discovered, our attention was drawn to the observation that about three quarters of the mutations identified were truncating mutations. Consequently, a truncated neurofibromin with a partial or absolute functional loss may be produced, or the protein may become degraded as a result of its abnormal termination, resulting in the inactivation of the negative regulatory protein. Therefore, the pathogenicity of the four novel truncating mutations that we identified is proverbially acknowledged because of a prematurely disrupted reading frame. Furthermore, we detected three different missense mutations that had been previously reported [21–26]. Nonetheless, multiple sequence alignment and in silico analysis were still performed to authenticate their pathogenicity (Additional file 1**:** Figure S3 and Table S2). Moreover, for Family 12 in whom a causative mutation was not found by either targeted next-generation sequencing or MLPA, we conjectured the existence of a deep intronic mutation.

Maruoka et al. [27] described a c.6853_6854insA (hg19; NM_001042492.2) *NF1* mutation that was identical to the mutation we found in Family 11 (Table 2). However, we thought a description of c.6791dupA (hg19; NM_000267.3) rather than c.6790_6791insA may be more appropriate in keeping with the ​standard human sequence variant nomenclature of the ​Human Genome Sequence Variation Society (HGVS) [28] and Mutalyzer website (version 2.0.23; Leiden University Medical Center, Leiden, the Netherlands) [29]. Likewise, the amino acid change in the c.1754_1757delTAAC mutation in Family 9 (Table 2) should be depicted as p.T586Vfs*18 [30], instead of p.L585fs*18 [31].

According to our observations, clinical manifestations in patients who had the same mutation within a family, and the same patient at different stages of their life, may be highly discrepant. Additionally, with regard to the polymorphisms in genotype–phenotype correlations, possible causes may be modifier genes, gender, or heterogeneity in the regulation of signaling pathways, to name a few.

Alterable though the phenotype of NF1 may be, the progression of NF1 was ascribed to age and pregnancy according to a long-term follow-up of several contactable patients. In general, it was older patients who usually manifested severer symptoms, while prepubertal patients displayed a comparatively mild phenotype with only café-au-lait spots. For example, Patient 7’s symptoms gradually worsened in the form of an increase in the number and size of cutaneous neurofibromas during a 10-year observation period (from 2007 to 2017; Fig. 2). Patients 1, 7, 16, 20 and 22 recalled that cutaneous neurofibromas appeared and symptoms became worse in their adolescence. Furthermore, some females with NF1 (Patients 1, 8, 10 and 18) complained of a marked exacerbation of disease after pregnancy (Fig. 2), as also described by Griffiths et al. [32]. These observations indicated that a patient’s physical condition, particularly their hormone levels, played a vital role in the development of NF1.

## Conclusions

Our research, via an integrated methodology, extends the *NF1* mutation spectrum in the Chinese population. Although a comprehensive investigation of the clinical profiles of patients was undertaken, we rarely found correlations between genotype and phenotype in NF1. Nevertheless, we noticed during follow-up observation that age and hormone levels were associated with the severity of disease.

## Additional file

Additional file 1: **Figure S1.** Pedigrees of families of sporadic cases. The arrows indicate the probands in each family. The asterisks denote that peripheral blood samples of individuals had been acquired. **a**–**e**: Pedigrees of Families 6–10. **Figure S2.** Multiplex ligation-dependent probe amplification (MLPA) results using P081 and P082 probemixes for patients in Family 12. **a**: MLPA results using P081 for Patient 22; **b**: MLPA results using P081 for Patient 23; **c**: MLPA results using P082 for Patient 22; **d**: MLPA results using P082 for Patient 23. **Figure S3.** Amino acid sequences of neurofibromin around missense mutations. Mutation sites are highlighted. **a**: The amino acid G629 and surrounding sequence; **b**: The amino acid L1490 and surrounding sequence; **c**: The amino acid W1931 and surrounding sequence. **Table S1.** Primers used in this study. **Table S2** In silico analysis of missense mutations. (PDF 781 kb)

## Abbreviations

*HGMD*: Human Gene Mutation Database
*HGVS*: Human Genome Sequence Variation Society
*IRB*: Institutional Review Board
*MLPA*: multiplex ligation-dependent probe amplification
*NCBI*: National Center for Biotechnology Information
*NF1*: neurofibromatosis type 1
*PAGE*: polyacrylamide gel electrophoresis
*PolyPhen-2*: Polymorphism Phenotyping v2
*RFLP*: restriction fragment length polymorphism
*SIFT*: Scale-Invariant Feature Transform
*SNP*: single nucleotide polymorphism
*SNV*: single nucleotide variant
*UCSC*: University of California, Santa Cruz
