# Supplementary material for: Clinical characteristics and spectrum of NF1 mutations in 12 unrelated Chinese families with neurofibromatosis type 1
Source: BMC Med Genet. 2018 Jun 18;19:101. doi: 10.1186/s12881-018-0615-8 (PMC6006597; doi:10.1186/s12881-018-0615-8)
Supplement: Supplementary file 1 — Figure S1. Pedigrees of families of sporadic cases. The arrows indicate the probands in each family. The asterisks denote that peripheral blood samples of individuals had been acquired. a–e: Pedigrees of Families 6–10. Figure S2. Multiplex ligation-dependent probe amplification (MLPA) results using P081 and P082 probemixes for patients in Family 12. a: MLPA results using P081 for Patient 22; b: MLPA results using P081 for Patient 23; c: MLPA results using P082 for Patient 22; d: MLPA results using P082 for Patient 23. Figure S3. Amino acid sequences of neurofibromin around missense mutations. Mutation sites are highlighted. a: The amino acid G629 and surrounding sequence; b: The amino acid L1490 and surrounding sequence; c: The amino acid W1931 and surrounding sequence. Table S1. Primers used in this study. Table S2 In silico analysis of missense mutations. (PDF 781 kb) [file 12881_2018_615_MOESM1_ESM.pdf]

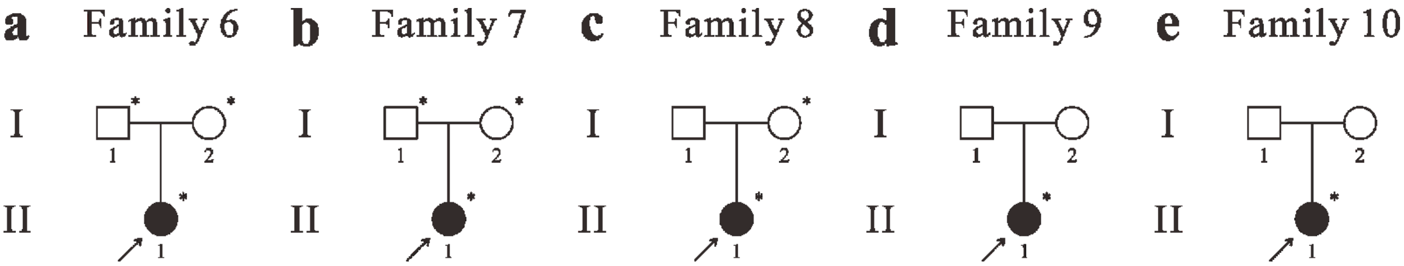

Fig. S1 Pedigrees of families of sporadic cases. The arrows indicate the probands in each family. The asterisks denote that peripheral blood samples of individuals had been acquired. **a**–**e**: Pedigrees of Families 6–10

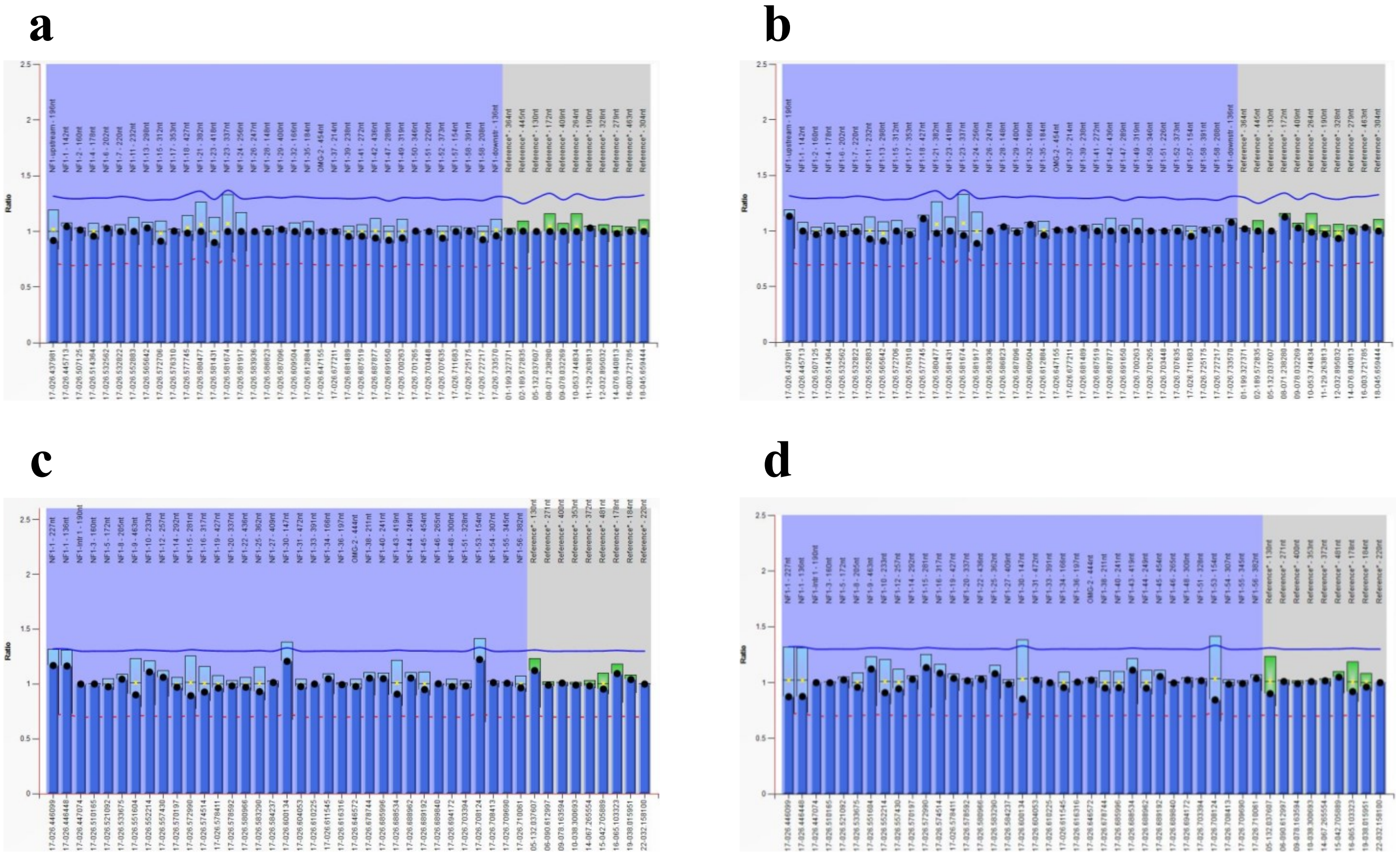

Fig.S2 Multiplex ligation-dependent probe amplification (MLPA) results of P081 and P082 for patients in Family 12. **a**: MLPA results of P081 for Patient 22; **b**: MLPA results of P081 for Patient 23; **c**: MLPA results of P082 for Patient 22; **d**: MLPA results of P082 for Patient 23

a 629

```
           ***** *
     Human KFLLKNKQADRSSCHFLLFYGVGCDIPSSGNTSQMSMDHEE
Chimpanzee KFLLKNKQADRSSCHFLLFYGVGCDIPSSGNTSQMSMDHEE
    Rhesus KFLLKNKQADRSSCHFLLFYGVGCDIPSSGNTSQMSMDHEE
       Bat KFLLKNKQADRSS--FLFLYGGGCDVPSSGNTSQMSMDHEE
       Dog KFLLKNKQADRSSCHFLFLYGVGCDVPSSGTTSQMSMDHEE
    Rabbit KFLLKNKQADRSSCHFLYLYGVGCDVPSSGNTSQMSMDHEE
     Mouse KFLLKNKQADRSSCHSLYLYGVGCEMSATGNTTQMSVDHDE
      Goat KFLLKNKQADRSSCHFLIVYGVGCDVPSSGNTSQMSMDHDE
     Horse KFLLKNKQADRSSCHFLFLYGVGCDVPSSGNTSQMSMDHEE
   Chicken KFLLKNKQSDRTSCHFLFLYDV-----SGGGASQMSLDHEE
      Toad KFLLKYK----------------------------------
      Fish KFLLKNK----------------------------------
```

b 1490

```
           :*********:****************:**************
     Human AVNHSLSFISDGNVLALHRLLWNNQEKIGQYLSSNRDHKAV
Chimpanzee AVNHSLSFISDGNVLALHRLLWNNQEKIGQYLSSNRDHKAV
    Rhesus AVNHSLSFISDGNVLALHRLLWNNQEKIGQYLSSNRDHKAV
       Bat AVNHSLSFISDGNVLALHRLLWNNQEKIGQYLSSNRDHKAV
       Dog TVNHSLSFISDGNVLALHRLLWNNQEKIGQYLSSNRDHKAV
    Rabbit AVNHSLSFISDGNVLALHRLLWNNQEKIGQYLSSNRDHKAV
     Mouse AVNHSLSFISDGNVLALHRLLWNNQEKIGQYLSSNRDHKAV
      Goat AVNHSLSFISDGNVLALHRLLWNNQEKIGQYLSSNRDHKAV
     Horse AVNHSLSFISDGNVLALHRLLWNNQEKIGQYLSSNRDHKAV
   Chicken TVNHSLSFISDGNVLALHRLLWNNQEKIGQYLSSNRDHKAV
      Toad TVNHSLSFITDGNVLALHRLLWNNQEKIGQYLSSNRDHKAV
      Fish SVNHSLSFISDGNVLALHRLLWNNQERIGQYLSSNRDHKAV
```

c 1931

```
           ********************** ******* .********:
     Human SGFSKSSIELKHLCLEYMTPWLSNLVRFCKHNDDAKRQRVT
Chimpanzee SGFSKSSIELKHLCLEYMTPWLSNLVRFCKHNDDAKRQRVT
    Rhesus SGFSKSSIELKHLCLEYMTPWLSNLVRFCKHNDDAKRQRVT
       Bat SGFSKSSIELKHLCLEYMTPWLSNLVRFCKHNDDAKRQRVT
       Dog SGFSKSSIELKHLCLEYMTPWLSNLVRFCKHNDDAKRQRVT
    Rabbit SGFSKSSIELKHLCLEYMTPWLSNLVRFCKHNDDAKRQRVT
     Mouse SGFSKSSIELKHLCLEYMTPWLSNLVRFCKHNDDAKRQRVT
      Goat SGFSKSSIELKHLCLEYMTPWLSNLVRFCKHNDDAKRQRVT
     Horse SGFSKSSIELKHLCLEYMTPWLSNLVRFCKHNDDAKRQRVT
   Chicken SGFSKSSIELKHLCLEYMTPWLSNLVRFCKHNDDAKRQRVT
      Toad SGFSKSSIELKHLCLEYMTPWLLNLVRFCKLTDDAKRQRVS
      Fish SGFSKSSIELKHLCLEYMTPWLLNLVRFCKHNDDAKRQRVT
```

Fig. S3 Amino acid sequences of neurofibromin around missense mutations. Mutation sites are highlighted. **a**: The amino acid G629 and surrounding sequence; **b**: The amino acid L1490 and surrounding sequence; **c**: The amino acid W1931 and surrounding sequence

Table S1 Primers used in this study

Application	Amplicon	Sequence (5'–3')	T_m (°C)	Size (bp)
Sanger	*NF1*-E9–11	F: GAGTTTTAGAGGCTGTTAATTTGC	55	2076
Sequencing		R: GGCAAATGAGGTGAAGGGTT	60	
	NF1-E16	F: GTCTCTGCTGTGCATGTGGTTTATT	59	752
		R: ACAATATGCTCCTCAAGCTACTCTG	59	
	NF1-E17	F: TAATGAATTCATAGTGCCCTGGTAG	57	969
		R: TGGGTTTTTTATGGAGTGGAG	54	
	NF1-E18	F: AGTTCTTGTTTATGCCCTTGTTTC	55	776
		R: TGTGCTTTGAGGCAGACTGAGTA	58	
	NF1-E28–29	F: CTTTGGGTTTACATTTTTGCTACTC	56	897
		R: ATTACCTATTAGCCTCTTTCCCTTC	57	
	NF1-E33–34	F: GCATAGGATTCATATTCTGGCACT	57	2328
		R: CATTAACCTTTATGAGGGTTCTG	55	
	NF1-E36	F: CAGGGTTCCAGTTTGTCTATTTC	57	1528
		R: CATATCCTCCACAGGACCTCCAT	60	
	NF1-E37	F: TTAGGCTGGAAGTAGAAGAGTGT	57	1521
		R: GAGTGATGTAGGTGGAAAGTATG	57	
	NF1-E39	F: GTTCTTTCTTCGCCTCTACAA	54	439
		R: GCTTTGCCCAATGTGGCACC	60	
	NF1-E40–41	F: TTCATATTGATTAGGCTGTTCC	53	919
		R: CACTGAGTATTCCGCTTATGG	56	
	NF1-E45	F: TCCGAGATTCAGTTTAGGAGTT	54	580
		R: ATCCATTCACTCCCCAACCT	55	
Mutation	*NF1*-Family 1	F: TTCTCATAGAAATAATCTGCTT	49	131
Validation		R: CCTTTTGGACTTACATTGGT	56	
	NF1-Family 2	F: ACTGGTCAAATCAATGGTGATA	53	101
		R: GTATGGGTAAGGTCCACTACAA	56	
	NF1-Family 3	F: AAGCCATTGTCCAGTCTATCA	54	151
		R: CTCGGGTCAGAACTGCCTAA	62	

Abbreviation: T_m: melting temperature calculated by Primer Premier 5 of the primer; E: exon; F: forward; R: reverse.

Table S2 *In silico* analysis of missense mutations

Mutation Position		Exon 17	Exon 33	Exon 39
Nucleotide Change		c.1885G>A	c.4469T>C	c.5791T>C
Amino Acid Change		p.G629R	p.L1490P	p.W1931R
UCSC Genome Browser	dbSNP 147	rs199474738	-	rs199474791
	Allele Frequencies	A: 0.002% (2 / 121202); G: 99.998% (121200 / 121202)		-
ExAC	Variant	17:29552152 G/A		
	Allele Count	2		
	Allele Number	121202		
	Number of Homozygotes	0		
	Allele Frequency	0.0000165		
Prediction Tool	PolyPhen-2	Probably Damaging	Probably Damaging	Probably Damaging
	SIFT	Tolerated	Affect Protein Function	Affect Protein Function
	Mutation Taster	Disease Causing	Disease Causing	Disease Causing
